# The Clinical Significance and Potential Molecular Mechanism of *PTTG1* in Esophageal Squamous Cell Carcinoma

**DOI:** 10.3389/fgene.2020.583085

**Published:** 2021-01-22

**Authors:** Shang-Wei Chen, Hua-Fu Zhou, Han-Jie Zhang, Rong-Quan He, Zhi-Guang Huang, Yi-Wu Dang, Xia Yang, Jun Liu, Zong-Wang Fu, Jun-Xian Mo, Zhong-Qing Tang, Chang-Bo Li, Rong Li, Li-Hua Yang, Jie Ma, Lin-Jie Yang, Gang Chen

**Affiliations:** ^1^Department of Cardio-Thoracic Surgery, The First Affiliated Hospital of Guangxi Medical University, Nanning, China; ^2^Department of Pathology, The First Affiliated Hospital of Guangxi Medical University, Nanning, China; ^3^Department of Medical Oncology, First Affiliated Hospital of Guangxi Medical University, Nanning, China; ^4^Department of Cardio-Thoracic Surgery, The Seventh Affiliated Hospital of Guangxi Medical University/Wuzhou Gongren Hospital, Wuzhou, China; ^5^Department of Pathology, Wuzhou Gongren Hospital/The Seventh Affiliated Hospital of Guangxi Medical University, Wuzhou, China

**Keywords:** *PTTG1*, esophageal squamous cell carcinoma, transcription factor, RNA sequencing, tissue microarray

## Abstract

Esophageal squamous cell carcinoma (ESCC) is the major histological type of esophageal cancers worldwide. Transcription factor *PTTG1* was seen highly expressed in a variety of tumors and was related to the degree of tumor differentiation, invasion, and metastasis. However, the clinical significance of *PTTG1* had yet to be verified, and the mechanism of abnormal *PTTG1* expression in ESCC was not clear. In this study, the comprehensive analysis and evaluation of *PTTG1* expression in ESCC were completed by synthesizing in-house immunohistochemistry (IHC), clinical sample tissue RNA-seq (in-house RNA-seq), public high-throughput data, and literature data. We also explored the possible signaling pathways and target genes of *PTTG1* in ESCC by combining the target genes of *PTTG1* (displayed by ChIP-seq), differentially expressed genes (DEGs) of ESCC, and *PTTG1*-related genes, revealing the potential molecular mechanism of *PTTG1* in ESCC. In the present study, *PTTG1* protein and mRNA expression levels in ESCC tissues were all significantly higher than in non-cancerous tissues. The pool standard mean difference (SMD) of the overall *PTTG1* expression was 1.17 (95% CI: 0.72–1.62, *P* < 0.01), and the area under curve (AUC) of the summary receiver operating characteristic (SROC) was 0.86 (95% CI: 0.83–0.89). By combining the target genes displayed by ChIP-seq of *PTTG1*, DEGs of ESCC, and *PTTG1*-related genes, it was observed that *PTTG1* may interact with these genes through chemokines and cytokine signaling pathways. By constructing a protein–protein interaction (PPI) network and combining ChIP-seq data, we obtained four *PTTG1* potential target genes, *SPTAN1*, *SLC25A17*, *IKBKB*, and *ERH*. The gene expression of *PTTG1* had a strong positive correlation with *SLC25A17* and *ERH*, which suggested that *PTTG1* might positively regulate the expression of these two genes. In summary, the high expression of *PTTG1* may play an important role in the formation of ESCC. These roles may be completed by *PTTG1* regulating the downstream target genes *SLC25A17* and *ERH*.

## Introduction

Esophageal cancer is the sixth leading cause of cancer-related deaths in the world and the eighth most common cancer globally; esophageal squamous cell carcinoma (ESCC) is the major histological type of esophageal cancers worldwide (Arnal et al., 2015). Despite the improved management and treatment of ESCC patients, the overall 5-year survival rate (10%) and 5-year survival rate following resection (15–40%) have remained low (Huang and Yu, 2018). The poor prognosis and rising morbidity of ESCC highlights the importance of improving detection methods, which is essential before treatment. As early clinical symptoms are not obvious, many ESCC patients are not diagnosed until the clinical symptoms appear in the late stage. In order to improve the prognosis of patients after surgery, patients were often treated with surgery combined with radiotherapy and chemotherapy, but this has not significantly increased the survival rate of ESCC patients. Since we have failed to fully understand the mechanism of the occurrence and development of ESCC, it is necessary and meaningful to explore its potential molecular mechanism to identify new prognostic biomarkers and potential ESCC therapeutic targets.

In recent years, studies have shown that transcription factors might play an important role in the occurrence and development of tumors, which has prompted us to study the clinical value and mechanism of transcription factor *PTTG1* in ESCC. *PTTG1* was the first proto-oncogene detected from rat pituitary tumor cells, encoding the securin protein—a protein that prevented separins from promoting the separation of sister chromatids (Pei and Melmed, 1997; Arenas et al., 2018). *PTTG1* was seen highly expressed in a variety of tumors and was related to the degree of tumor differentiation, invasion, and metastasis (Vlotides et al., 2007). It was shown that *PTTG1* played an essential role in the biological function of tumor cells. Its high expression in colorectal cancer promoted proliferation and metastasis (Ren and Jin, 2017). Its high expression in hepatocellular carcinoma, small cell lung carcinoma, and prostate cancer had value for clinical diagnosis and prognosis (Kong et al., 2019; Yang et al., 2019; Ersvær et al., 2020). Although studies found that *PTTG1* was likely to exhibit high expression in ESCC, the number of cases has been small and the research was based on a single center study, using a single detection method (Shibata et al., 2002; Ito et al., 2008; Feng et al., 2017). There have been only two reports on the mechanism of *PTTG1* in ESCC. It was found that *PTTG1* promoted the movement and migration of ESCC cells by activating related genes (Ito et al., 2008; Feng et al., 2017). The possible target genes of *PTTG1* as a transcription factor in ESCC had not been reported yet, and the practice of using integrated computational biology to mine ChIP-seq data to study the underlying molecular mechanism of *PTTG1* had not been reported.

Based on the existing research on *PTTG1* in ESCC, this study used clinical samples to make tissue chips, performed immunohistochemistry (IHC) to detect the expression level of *PTTG1* protein in ESCC tissues, and used our clinical sample tissues to conduct RNA sequencing (RNA-seq) to verify the expression of *PTTG1* mRNA. The comprehensive analysis and evaluation of *PTTG1* expression in ESCC were completed by synthesizing in-house IHC, clinical sample tissue RNA-seq (in-house RNA-seq), public high-throughput data, and literature data. We also explored the possible signaling pathways and target genes of *PTTG1* in ESCC by combining the target genes of *PTTG1* (displayed by ChIP-seq), differentially expressed genes (DEGs) of ESCC, and *PTTG1*-related genes (Supplementary Figure 1), revealing the potential molecular mechanism of *PTTG1* in ESCC and providing a new target for ESCC research.

## Materials and Methods

### Expression Level of *PTTG1* Protein in Tissue Chip

A total of 302 cases were collected, including 159 ESCC tissues and 143 paracancerous tissues of 153 males and 18 females with a median age of 58 years. These chips (ESC242, ESC1503, and ESC1504) were produced by Fanpu Biotech, Inc. (Guilin, China). Two experienced pathologists read each case separately. *PTTG1* was a product of Biorbyt (Catalog No. orb3740037, Cambridge, United Kingdom). IHC was performed according to the instructions. The results were scored according to the IRS scoring system of 0–12 points (Lin et al., 2018). The scores of staining intensity were divided into 0, 1, 2, and 3, which represented negative, weak, medium, and strong, respectively. Positive staining proportion was classified as 0 for <10%, 1 for 11–25%, 2 for 26–50%, 3 for 51–75%, and 4 for 76–100%. The ultimate score was obtained based on the product of the positive proportion and the staining intensity. Then, the relationship between the *PTTG1* protein expression level and clinicopathological parameters was analyzed based on the IHC score.

### RNA-Seq of Clinical Samples

We collected eight pairs of ESCC samples and corresponding adjacent normal esophageal tissues for RNA-seq from eight ESCC patients who underwent radical resection of EC from the First Affiliated Hospital of Guangxi Medical University (Nanning, Guangxi, China), from April to August 2019. None of these ESCC patients received preoperative chemotherapy or radiotherapy. The tissue samples were stored at −80°C immediately after resection. These eight patients included six males and two females with a median age of 60 years. RNA-seq was executed by Wuhan Seqhealth Technology Co., LTD. (Wuhan, China). TRIzol reagent (Invitrogen, Carlsbad, CA, United States) was applied to extract and prepare total RNA from tumors and paired adjacent non-ESCC tissues. A total amount of 3 μg RNA per sample was used as initial material for the RNA sample preparations. Nanodrop^TM^ 1000 spectrophotometer (Thermo Fisher Scientific, United States) was applied to examine RNA purity. RNA concentration was determined using a Qubit^®^ 3.0 Fluorometer (Life Technologies, CA, United States). RNA integrity was determined using an RNA Nano 6000 Assay Kit and a Bioanalyzer 2100 system (Agilent Technologies, CA, United States). Afterward, the sequencing libraries were established using the NEBNext Ultra Directional RNA Library Prep Kit for Illumina (NEB, Ipswich, MA, United States) based on manufacturer’s protocols. The 200 Illumina HiSeq X Ten platform was used to sequence the libraries. RNA-seq reads were aligned to the reference of homo_sapiens. GRCh38.^1^ In the current study, we obtained the data on log2 (x + 0.001) conversion level 3 and reported RNA-seq data in transcripts per kilobase million (TPM). All enrolled patients signed an informed consent form, and the research including tissue chip and RNA-seq was approved by the ethics committee of the First Hospital Affiliated to Guangxi Medical University.

### Collection of High-Throughput Data Sets

In order to compare the differences in gene expression between ESCC tissues and non-cancerous esophageal tissues, we used Gene Expression Omnibus (GEO), SRA, ArrayExpress, Oncomine, The Cancer Genome Atlas (TCGA), and various literature databases to extensively search ESCC-related data sets. The search formula is (Esophageal OR Esophagus) AND (maliganan^∗^ OR cancer OR tumor OR neoplas^∗^ OR carcinoma), AND “Homo sapiens” (porgn:_txid9606). All the information, including summary and overall design, of these data sets was then further evaluated.

### Statistical Analysis

Student’s *t*-test was performed using SPSS 22.0 to compare the differential expression of *PTTG1* between ESCC and non-cancerous tissues. The calculated results were presented in the form of the mean ± standard deviation (SD). GraphPad Prism 8.0 was applied to draw scatter plots and box plots. The area under curve (AUC) of receiver operating characteristic (ROC) was used to evaluate the ability of *PTTG1* levels to distinguish ESCC tissues from non-cancer tissues. In order to systematically analyze the expression level of *PTTG1*, we comprehensively analyzed in-house IHC, in-house RNA-seq, public RNA-seq, gene chip data, and published data. The standard mean difference (SMD) and 95% confidence interval (CI) were calculated by R 3.6.1, and the expression levels of *PTTG1* in ESCC and non-cancer tissues were compared. The funnel chart was employed to detect publication bias. Synthesizing all the data, the researchers used Stata12.0 to calculate the summary receiver operating characteristic (SROC) and further clarified the differential expression of *PTTG1* in ESCC and non-cancer tissues. Student’s *t*-test was used to show the heterogeneity of the results. When *P* < 0.05 or *I*^2^ > 50%, there was heterogeneity, and a random effect model was used. When *P* > 0.05 or *I*^2^ < 50%, no obvious heterogeneity was detected, and a fixed-effect model was used. *P* < 0.05 was considered statistically significant.

### Screening for *PTTG1* Target Genes, Esophageal Cancer Differentially Expressed Genes and *PTTG1*-Related Genes

Since *PTTG1* was a transcription factor, in order to obtain the key downstream target genes of *PTTG1*, we collected *PTTG1* target genes and potential binding sites proved by ChIP-seq through the Cistrome DB database^2^ (Mei et al., 2017). Subsequently, we used the LIMMA package to process all gene chips containing *PTTG1* expression data and used LIMMA-VOOM to process DEGs of RNA-seq data [| log fold change (FC)| > 1, *P* < 0.05]. By calculating the correlation coefficient, the related genes were calculated in each high-throughput data set including *PTTG1* (| *r*| > 0.5, *P* < 0.05). Because each of the above steps contained multiple data sets, we researchers extracted genes that met the criteria in all data sets for intersection.

### Functional Enrichment Analysis and Construction of Protein–Protein Interaction Networks to Screen Key Target Genes

In order to study the possible functions of these key target genes, the researchers carried out gene ontology (GO) functional enrichment analysis and Kyoto Encyclopedia of Genes and Genomes (KEGG) pathway enrichment analysis and visualization. We applied Cytoscape 3.7.1 to construct a protein–protein interaction (PPI) network of these genes and then used the MCODE algorithm to determine the hub gene of the protein densely connected neighborhood. The University of California, Santa Cruz (UCSC) Genome Browser on Human (GRCh38/hg38) Assembly was employed to display the binding position of the target gene and *PTTG1* in ChIP-seq.

## Results

### Expression Level of *PTTG1* Protein in Tissue Microarray

**FIGURE 1:**
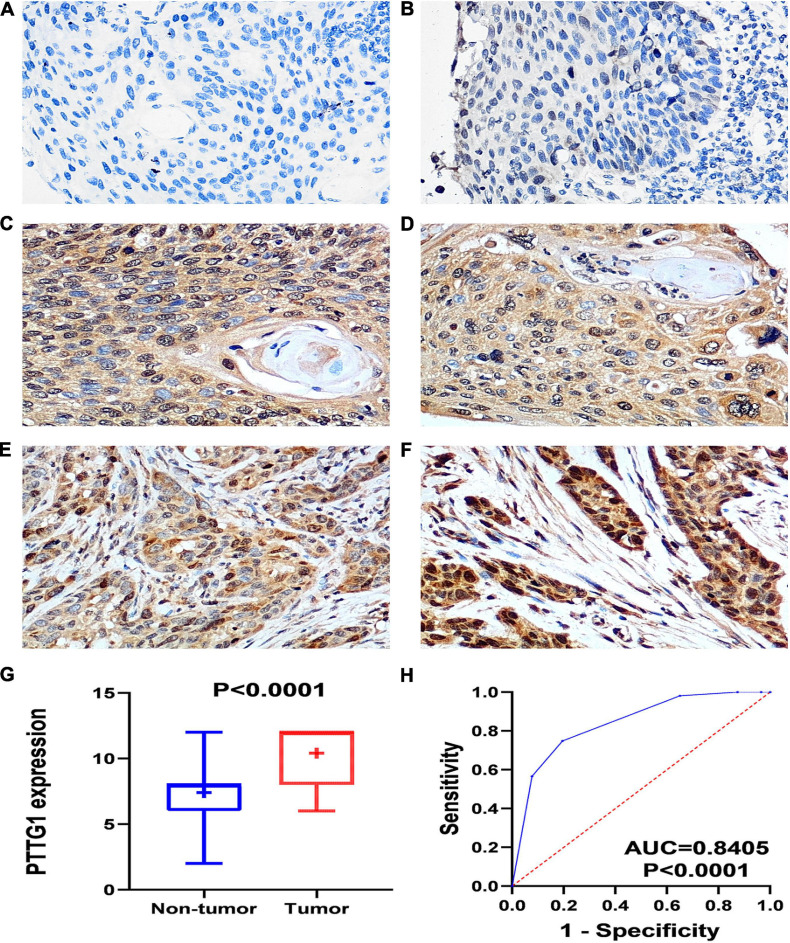
*PTTG1* protein expression elevated in esophageal squamous cell carcinoma (ESCC) tissue chip assessed by in-house immunohistochemistry. **(A,B)** Immunohistochemical staining of *PTTG1* protein in non-cancerous esophageal tissues from different cases. Both cases were scored as 0 (×400); **(C–F)** ESCC tissues (×400). The scores of case **(C)** and case **(D)** were both 6. The score was recorded as 8 for case **(E)** and 12 for case **(F)**; **(G)** box diagram; **(H)** receiver operating characteristic (ROC) curve; AUC, area under the curve.

The IHC scores of *PTTG1* in each ESCC and normal case based on two pathologists were presented in Supplementary Table 1. The intraclass correlation coefficients of IHC score in ESCC and normal tissues between two pathologists were 0.943 and 0.938, respectively. IHC staining of tissue chips showed that the *PTTG1* protein expression level in ESCC tissues was significantly higher than in non-cancerous tissues (10.410 ± 1.880 vs. 7.410 ± 2.153, *P* < 0.0001; AUC = 0.8405, *P* < 0.0001) (Figure 1). However, *PTTG1* expression did not show a significant correlation with various clinical parameters (Supplementary Table 2).

### Expression Level of PTTG1 mRNA in In-House RNA-Seq

**FIGURE 2:**
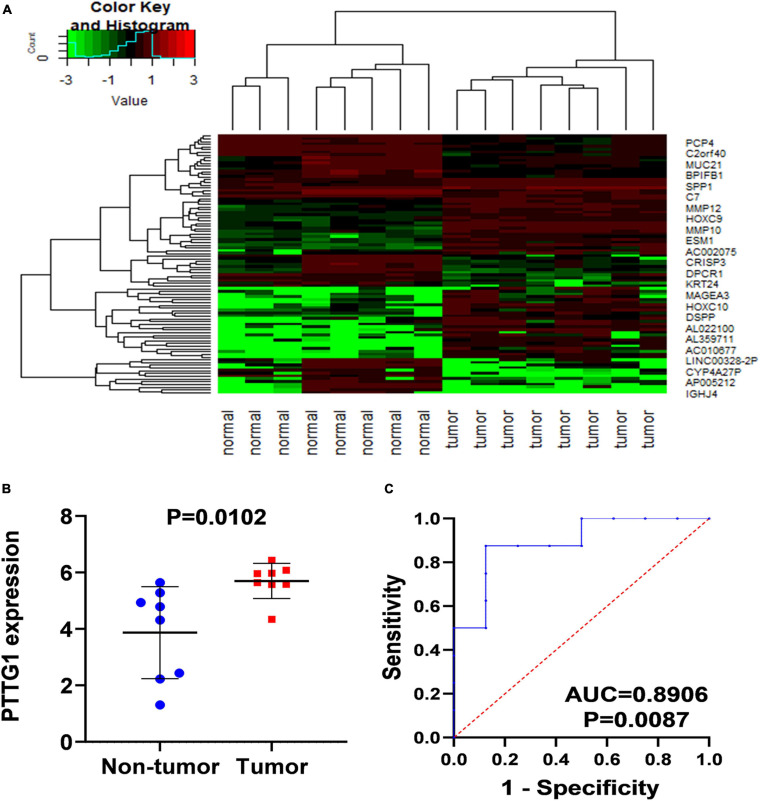
In-house RNA-seq confirmed *PTTG1* overexpression in ESCC tissues. **(A)** Heatmap of differentially expressed genes (DEGs). **(B)** Scatter plot. **(C)** ROC curve.

In order to confirm the high expression of *PTTG1* in ESCC tissues, we collected ESCC tissues and their matched non-cancer tissues from eight clinical patients with ESCC and obtained in-house RNA-seq data of these samples. It was found that the expression of *PTTG1* in ESCC tissues was significantly higher than in the adjacent tissues (5.6999 ± 0.6218 vs. 3.8690 ± 1.6307, *P* = 0.0102) (Figures 2A,B). ROC analysis showed that the expression of *PTTG1* was remarkably different between ESCC and non-cancerous tissues (AUC = 0.8906, *P* = 0.0087) (Figure 2C). These results further confirmed that *PTTG1* was overexpressed in ESCC.

### Clinical Significance of *PTTG1* Expression Based on Public High-Throughput Data

**FIGURE 3:**
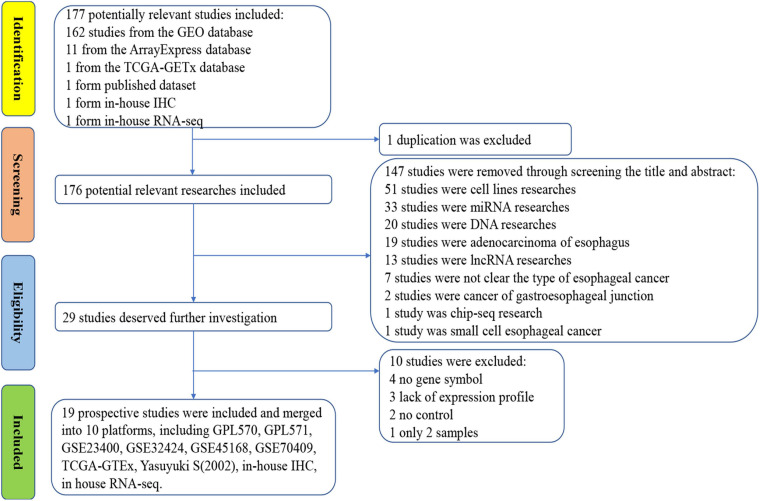
Flow chart of data collection for this study.

**FIGURE 4:**
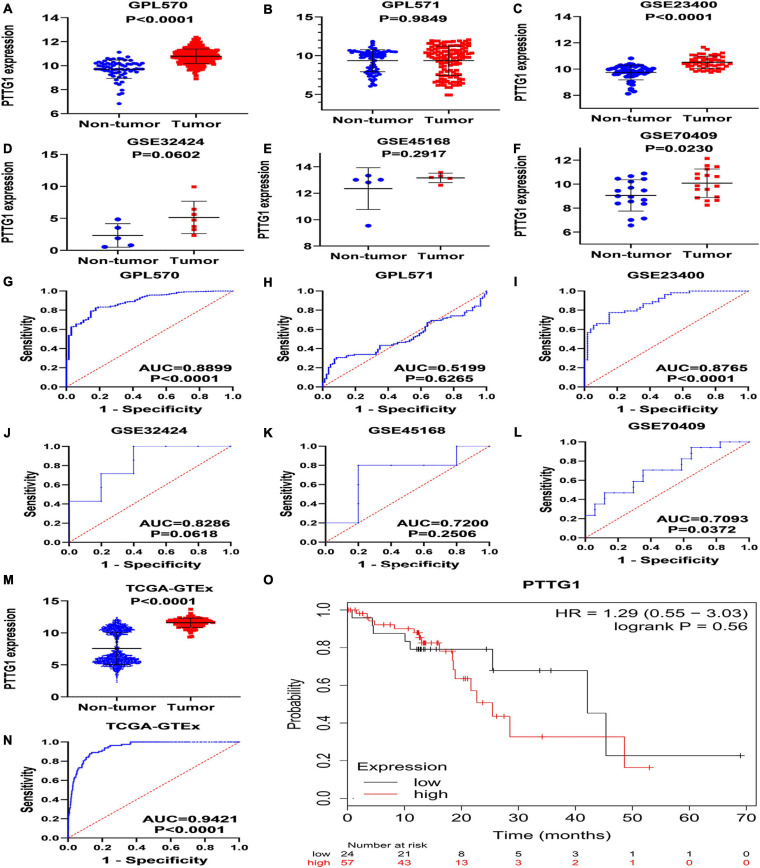
Expression of *PTTG1* mRNA upregulates in ESCC based on high-throughput RNA-seq of public database. GPL570 **(A,G)**, GPL571 **(B,H)**, GSE23400 **(C,I)**, GSE32424 **(D,J)**, GSE45168 **(E,K)**, and GSE70409 **(F,L)**. The cancer genome atlas (TCGA) dot plot and ROC curve **(M–O)** KM survival analysis curve based on TCGA data from KMplot database; the median expression value of *PTTG1* was applied as cut-off value to classify patients into low and high expression groups.

To verify the results of our immunohistochemistry and RNA-seq experiments, a total of 15 GeneChips of ESCC tissues were gathered from public databases, namely, GSE45670GPL570, GSE77861GPL570, GSE26886GPL570, GSE69925GPL570, GSE100942GPL570, GSE33810GPL570, GSE17351GPL570, GSE20347GPL571, GSE38129GPL571, GSE29001GPL571, GSE33426GPL571, GSE23400GPL96, GSE32424, GSE45168, and GSE70409. In order to avoid batch effects, the GPL570 and GPL571 from the same platform were combined. In order to improve the reliability of differential mRNA screening results, we downloaded the RNA-seq data and clinical follow-up information of ESCC. All data collection processes are shown in Figure 3. We used seven data sets, including six GeneChips (GPL570, GPL571, GSE23400, GSE32424, GSE45168, and GSE70409), and RNA-seq data (2,307 cases in total, with 620 cases of ESCC and 1,687 non-cancer cases) to investigate the expression of *PTTG1* in ESCC. After research, it was found that the expression of *PTTG1* in four high-throughput platforms (GPL570, GSE23400, GSE70409, and TCGA-GTEx) was significantly higher than in the non-cancer control group (Figures 4A–F,M). ROC analysis of all data sets showed that the expression of *PTTG1* had different degrees of significance for the clinical diagnosis of ESCC (Figures 4G–L,N). Further analysis of public RNA-seq data showed that the average expression of *PTTG1* in ESCC was 11.5794 ± 0.7270, which was significantly higher than that of the non-cancer control group (7.5575 ± 2.5127, *P* < 0.001). We also analyzed the relationship between the expression of *PTTG1* and the clinical pathological parameters of ESCC patients. The expression of *PTTG1* was negatively correlated with the age of ESCC patients (*r* = −0.3097, *P* = 0.004), and the expression of *PTTG1* in young patients was higher (11.8 ± 0.7 vs. 11.3 ± 0.6, *P* = 0.004) (Supplementary Table 3). However, no significant difference in *PTTG1* expression was found in other clinicopathological parameters. The survival condition of patients with high *PTTG1* expression was worse than that of patients with low *PTTG1* expression (HR = 1.29), but no significant statistical difference was found (Figure 4O).

### Comprehensive Analysis of *PTTG1* Expression

**FIGURE 5:**
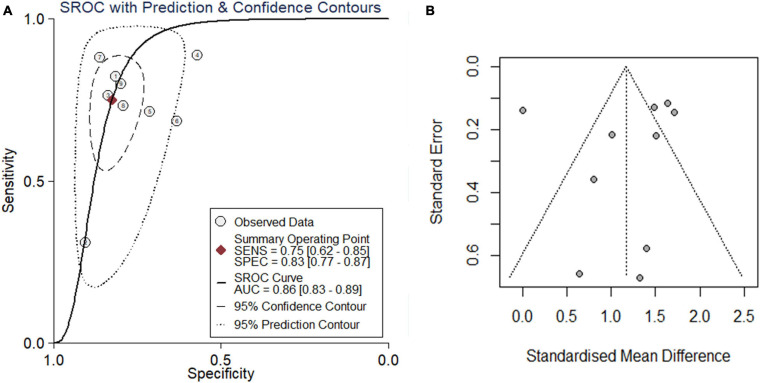
Comprehensive analysis further confirmed that PTTG1 was significantly overexpressed in ESCC. **(A)** Summary receiver operating characteristic (SROC) curve. **(B)** Funnel chart of publication bias. AUC, area under curve.

Because the research scale of a single data set was too small, it was difficult to draw effective conclusions. To make the results more reliable, we combined public RNA-seq, GeneChip, in-house IHC, in-house RNA-seq data, and published data (Shibata et al., 2002) for analysis. Our research included 835 cancer tissues and 1,886 non-cancer tissues. Considering the heterogeneity of our data, we chose a random effects model. The SMD of the overall *PTTG1* expression was 1.17 (95% CI: 0.72–1.62, *P* < 0.01, Table 1), which indicated that *PTTG1* was noticeably expressed in ESCC. The area under the SROC curve was 0.86 (95% CI: 0.83–0.89), with sensitivity being 0.75 (95% CI: 0.62–0.85), and specificity being 0.83 (95% CI: 0.77–0.87) (Figure 5A and Supplementary Figure 2A). The positive likelihood ratio was 4.31 (95% CI: 3.21–5.80), and the negative likelihood ratio was 0.30 (95% CI: 0.19–0.48) (Supplementary Figure 2B). The diagnostic odds ratio was 14.22 (95% CI: 7.60–26.62) (Supplementary Figure 2C). To visualize the publication bias, we performed Begg’s funnel plot and Egger’s test. The Begg’s test showed no obvious publication bias (*P* = 0.175, Figure 5B). Egger’s test also showed that the high expression of *PTTG1* in ESCC did not have a publication bias (*P* = 0.844). After a comprehensive analysis, *PTTG1* was confirmed to display high expression in ESCC.

**TABLE 1:** Comprehensive analysis of *PTTG1* expression in esophageal squamous cell carcinoma (ESCC) based on gene microarrays, in-house immunohistochemistry (IHC), public RNA-seq, and in-house RNA-seq.

Study ID	ESCC			Normal			SMD	95% CI
	*n*	Mean	SD	*n*	Mean	SD		
GPL570	329	10.78	0.598	68	9.70	0.765	1.71	1.42; 2.00
GPL571	127	9.36	1.890	83	9.35	1.423	0.00	−0.27; 0.28
GSE23400	53	10.52	0.467	53	9.74	0.559	1.51	1.07; 1.94
GSE32424	7	2.52	0.566	5	1.54	0.829	1.32	0.00; 2.64
GSE45168	5	13.16	0.355	5	12.34	1.578	0.64	−0.65; 1.94
GSE70409	17	10.08	1.178	17	9.06	1.307	0.80	0.10; 1.50
TCGA-GTEx	82	11.58	0.727	1,456	7.56	2.512	1.64	1.41; 1.87
Yasuyuki S (2002)	48	0.10	0.068	48	0.05	0.041	1.01	0.58; 1.43
In-house IHC	159	10.41	1.880	143	7.41	2.153	1.49	1.23; 1.74
In-house RNA-seq	8	5.70	0.062	8	3.87	1.630	1.40	0.27; 2.53
Overall (*I*^2^ = 0.92, *P* < 0.01)	835			1,886			1.17	0.72, 1.62

*ESCC, esophageal squamous cell carcinoma; IHC, immunohistochemistry; *n*, number; SD, standard deviation; SMD, standard mean difference; CI, confidence interval.*

### Enrichment Analysis of Differentially Expressed Genes, *PTTG1*-Related Genes and Predicted Target Genes and Construction of Protein–Protein Interaction Network

**FIGURE 6:**
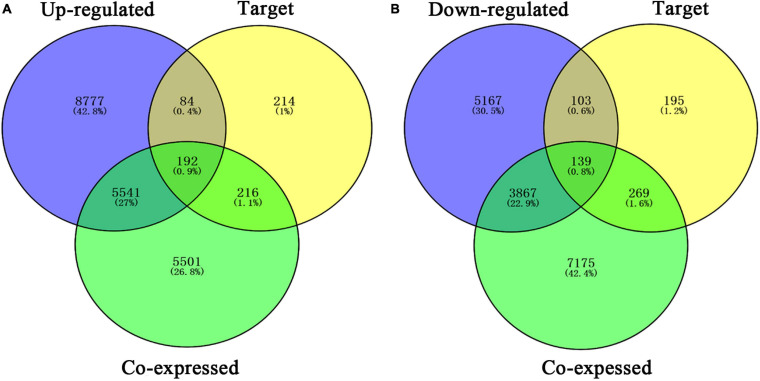
Venn diagrams of overlapping *PTTG1*-related genes, target genes, and upregulated genes **(A)** and downregulated genes **(B)**. Upregulated, upregulated differential genes; downregulated, downregulated differential genes; target, target genes of *PTTG1* in ChIP-seq.

**FIGURE 7:**
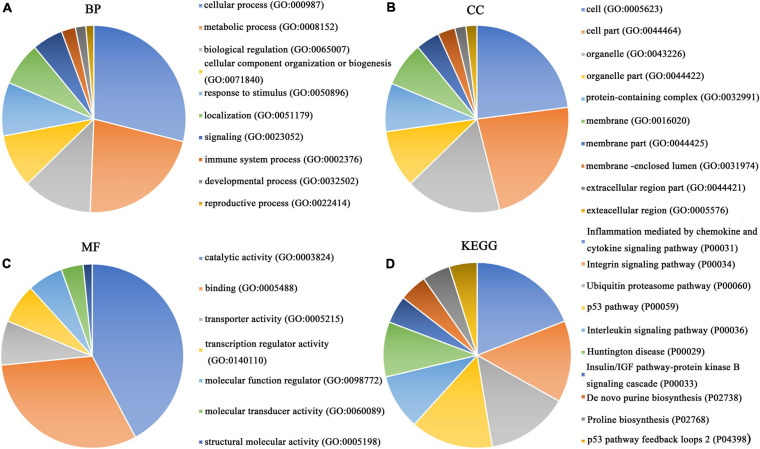
Gene ontology (GO) function annotation and Kyoto Encyclopedia of Genes and Genomes (KEGG) pathway analysis of upregulated overlapping genes. **(A)** Biological process. **(B)** Cell composition. **(C)** Molecular function. **(D)** KEGG pathway analysis.

**FIGURE 8:**
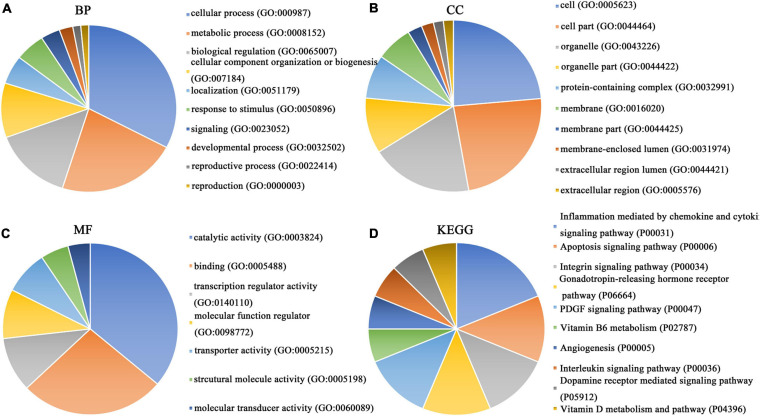
GO function annotation and KEGG pathway analysis of downregulated overlapping genes. **(A)** Biological process. **(B)** Cell composition. **(C)** Molecular function. **(D)** KEGG pathway analysis.

**FIGURE 9:**
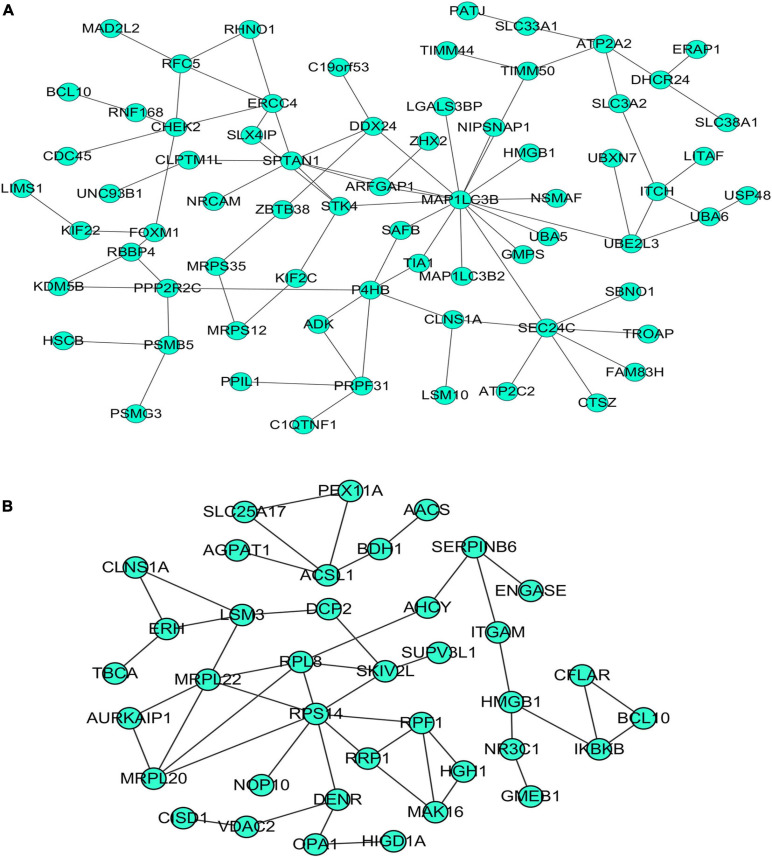
Protein–protein interaction (PPI) network of overlapping genes. **(A)** PPI network of upregulated overlapping genes. **(B)** PPI network of downregulated overlapping genes.

**FIGURE 10:**
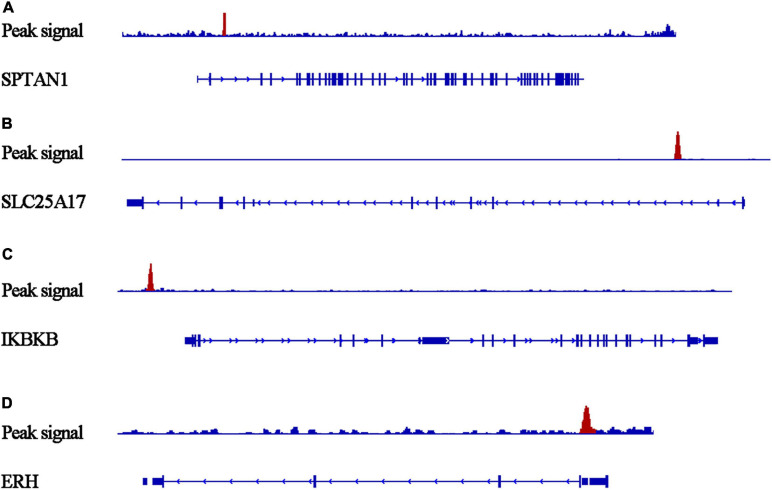
Peaks of target genes binding to *PTTG1* in ChIP-seq based on the Cistrome DB database. The peak signals of each target genes are colored red. **(A)** *SPTAN1*; **(B)** *SLC25A17*; **(C)** *IKBKB*; **(D)** *ERH*.

**FIGURE 11:**
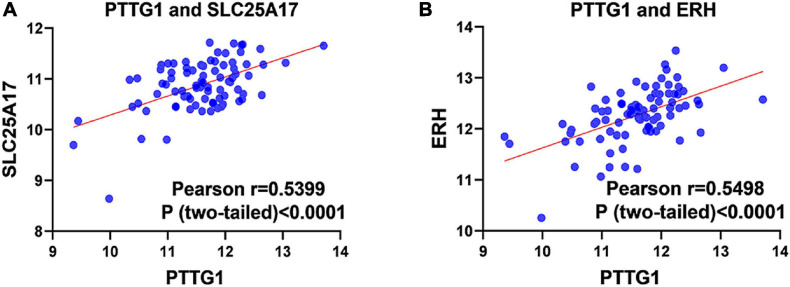
Pearson correlation analysis of *PTTG1* and its target genes. **(A)** Pearson correlation between *PTTG1* and *SLC25A17*. **(B)** Pearson correlation between *PTTG1* and *ERH*.

Our researchers obtained a total of 23,872 DEGs, including 14,594 upregulated genes, 9,278 downregulated genes, and 11,450 related genes from six GeneChip platforms, TCGA-GTEx RNA-seq and in-house RNA-seq. The Cistrome DB database was used to predict *PTTG1* target genes, and 19,719 genes were acquired. We selected all the *PTTG1*-related genes to combine with 706 target genes with a score greater than 0.5 in the results of the Bistro DB ChIP-seq. We examined intersections with the upregulated and downregulated genes, respectively, and eventually gained 192 genes that crossed with the upregulation and 139 genes that overlapped with downregulation (Figures 6A,B). Then, we conducted GO function enrichment, including biological process (BP), cellular component (CC), molecular function (MF), and KEGG pathway analysis. Figures 7, 8 illustrate the enrichment analysis of upregulated and downregulated overlapping genes, respectively. As shown in the figures, the results of gene enrichment of the two parts were similar. In the BP process, these genes were mainly enriched in cellular processes, metabolic processes, and biological regulation; in CC, they were largely enriched in organelles; in MF, they were mostly concentrated in functions such as catalytic activity, binding, and transportation activities; in KEGG pathway analysis, they were mainly enriched in inflammation mediated by chemokine and cytokine signaling pathway. The upregulated genes were also enriched in the ubiquitin proteasome pathway, while the downregulated genes were enriched in the apoptosis signaling pathway. This indicated that *PTTG1* may play a biological role in ESCC through these pathways. We also constructed a PPI network for these overlapping genes (Figure 9) and acquired the hub gene using MCODE. The hub genes we obtained were *ARFGAP1*, *UBA6*, *STK4*, *ERCC4*, *SPTAN1*, *DDX24*, *RFC5*, *CHEK2*, *MAP1LC3B*, *ITCH*, *UBE2L3*, *MRPL20*, *MRPL22*, *RPL8*, *RPS14*, *PEX11A*, *ACSL1*, *SLC25A17*, *IKBKB*, *CFLAR*, *BCL10*, *CLNS1A*, *LSM3*, and *ERH*. These hub genes were entered into the Cistrome DB website. In the ChIP-seq track, each block represented a peak where the target gene would bind to the corresponding transcription factor. Figure 10 shows that the *SPTAN1* and *PTTG1* binding peak could be seen at the site 278,308 bp away from the *PTTG1* transcription start site, *SLC25A17* was detected at 166,478 bp, *IKBKB* was discovered at 173,073 bp, and the overlapped peak of *ERH* and *PTTG1* was found at 96,965 bp. The transcription initiation sites of these genes were all integrated with *PTTG1* (Figure 10). To further explore the relationship between these target genes and *PTTG1*, we calculated the SMD of these genes and found that *SLC25A17* (SMD = 1.13, 95% CI: 0.04–2.22, *P* < 0.01; Table 2) and *ERH* (SMD = 1.20, 95% CI: 0.26–2.15, *P* < 0.01; Table 3) were significantly highly expressed. We also analyzed the correlation between *PTTG1* and these two target genes. The results showed that *PTTG1* and *SLC25A17* (*r* = 0.5399, *P* < 0.0001) (Figure 11A) and *ERH* (*r* = 0.5498, *P* < 0.0001) (Figure 11B) had a strong positive correlation, which suggested that *PTTG1* might positively regulate the expression of these two genes. These results indicated that *PTTG1* played an important part in regulating the functions of these proteins.

**TABLE 2:** Comprehensive analysis of *SLC25A17* expression in ESCC based on gene microarrays, public RNA-seq, and in-house RNA-seq.

Study ID	ESCC			Normal			SMD	95% CI
	*n*	Mean	SD	*n*	Mean	SD		
GPL570	329	630.1	247.1	68	649.4	104.5	−0.08	−0.34; 0.18
GPL571	127	176.6	188.6	83	253.9	169.1	−0.43	−0.70; 0.28
GSE23400	53	6.54	0.207	53	6.28	0.114	1.52	1.07; 1.94
GSE32424	7	3.14	0.172	5	1.56	0.532	4.02	1.75; 6.29
GSE45168	5	10.31	0.354	5	9.82	0.413	1.14	−0.25; 2.54
GSE70409	17	9.45	0.678	17	9.40	0.794	0.08	−0.60; 0.75
TCGA-GTEx	82	10.88	0.505	1,456	9.54	0.515	2.60	2.36; 2.84
In-house RNA-seq	8	4.19	0.498	8	3.64	0.348	1.21	0.12; 2.30
Overall (*I*^2^ = 0.98, *P* < 0.01)	628			1,695			1.13	0.04; 2.22

*ESCC, esophageal squamous cell carcinoma; *n*, number; SD, standard deviation; SMD, standard mean difference; CI, confidence interval.*

**TABLE 3:** Comprehensive analysis of *ERH* expression in ESCC based on gene microarrays, public RNA-seq, and in-house RNA-seq.

Study ID	ESCC			Normal			SMD	95% CI
	*n*	Mean	SD	*n*	Mean	SD		
GPL570	329	8,505	4,309	68	8,503	2,543	0.00	−0.26; 0.26
GPL571	127	3,905	4,576	83	5,676	3,759	−0.41	−0.69; −0.13
GSE23400	53	10.98	0.284	53	10.33	0.267	2.37	1.87; 2.87
GSE32424	7	6.58	0.356	5	5.22	0.358	3.52	1.46; 5.59
GSE45168	5	13.18	0.502	5	12.91	0.138	0.67	−0.63; 1.96
GSE70409	17	12.16	0.830	17	11.54	0.916	0.70	0.01; 1.40
TCGA-GTEx	82	12.27	0.533	1,456	11.27	0.495	2.00	1.77; 2.24
In-house RNA-seq	8	6.20	0.488	8	5.54	0.217	1.66	0.48; 2.84
Overall (*I*^2^ = 0.97, *P* < 0.01)	628			1,695			1.20	0.26; 2.15

*ESCC, esophageal squamous cell carcinoma; *n*, number; SD, standard deviation; SMD, standard mean difference; CI, confidence interval.*

## Discussion

Although some studies reported on the expression level of *PTTG1* in ESCC, its clinical significance had yet to be verified, and the mechanism of abnormal *PTTG1* expression in ESCC was not clear. To address these issues, this study used a variety of detection methods to investigate the expression level of *PTTG1* in ESCC, including in-house IHC, in-house RNA-seq, gene chips, public RNA-seq, and a published study. We conducted a comprehensive integrated analysis of the expression data of 2,721 samples from independent studies to calculate SMD and SROC. This study on *PTTG1* expression in ESCC had the largest sample size of any related study by far. The analysis results showed that the expression of *PTTG1* in ESCC was upregulated. As a transcription factor, *PTTG1* could target and regulate specific genes to perform biological functions. Combining the analysis results of DEGs, *PTTG1*-related genes of ESCC, and ChIP-seq of *PTTG1*, we found for the first time that *SLC25A17* and *ERH* were likely to be the target genes of *PTTG1* in ESCC. These findings could help to study the key role of *PTTG1* in the regulation mechanism in ESCC.

So far, there have been only three reports on the clinical significance of *PTTG1* expression in ESCC. The sample size of these was relatively small, and they were based on a single center study. The use of a single detection method failed to fully represent the role of *PTTG1* in ESCC. At the time of these reports, the widely used high-throughput technology had not been adopted. Differences existed among the results of several studies. One study using RT-PCR of 48 cases of ESCC and its matched samples revealed that *PTTG1* expression was upregulated in ESCC, and the average expression level in tumor tissue was 2.19 times than that of normal tissue (0.105 vs. 0.048) (Shibata et al., 2002). In another study, the immunohistochemistry of 50 ESCC samples and normal esophageal tissues suggested that *PTTG1* was highly expressed in ESCC. Of 50 ESCC tissues, 31 (62%) were positive, while only 17 of 50 normal tissues (34%) were positive (Feng et al., 2017). Another study, using 113 cases of ESCC immunohistochemistry and immunoblotting, revealed the overexpression of *PTTG1*. It was found that 14 tumors in 113 cases were negatively expressed; 31 cases expressed 0–10% of tumor cells (1+), 52 cases showed 10–30% tumor cell expression (2+), and 16 cases showed 30% tumor cell expression (3+). *PTTG1* expression of 2+ or 3+ was determined as *PTTG1* positive (68/113, 60.2%), while *PTTG1* staining of normal esophageal epithelium showed no intracytoplasmic staining and occasionally intranuclear staining (about 10%) (Ito et al., 2008). In order to expand the sample size and integrate various detection methods and multi-center data, in this study, we employed various detection methods (immunohistochemistry of tissue chips, RNA-seq, microarrays, and RT-PCR) to study the clinical significance of *PTTG1* expression in ESCC tissues. The single detection method and the comprehensive results indicated that the *PTTG1* protein and mRNA were highly expressed in ESCC, which was consistent with the reported results (Shibata et al., 2002; Ito et al., 2008; Feng et al., 2017). In the study, SMD = 1.17 (95% CI: 0.72–1.62, *P* < 0.01), and this result was strongly supported by the SROC combined with AUC 0.86 (95% CI: 0.83–0.89). This study used a large sample to verify the upregulation of *PTTG1* in ESCC, suggesting that high expression of *PTTG1* might promote the occurrence of ESCC.

In addition to its role in promoting ESCC, we also discussed the possibility of *PTTG1* advancing tumor progression. It was reported in the literature that the high expression of *PTTG1* was closely related to the pathological stage and lymph node metastasis (Shibata et al., 2002). *In vitro*, knocking down *PTTG1* in EC-1 and Eca-109 cell lines could inhibit EMT, migration, and metastasis (Ito et al., 2008; Feng et al., 2017). This study also examined the relationship between the progress of *PTTG1* and ESCC on a variety of data sets. In our study, we have confirmed that *PTTG1* was significantly overexpressed in ESCC tissues, and in the prognosis analysis, the survival condition of patients with high *PTTG1* expression was worse than that of patients with low *PTTG1* expression (HR = 1.29), but no significant statistical difference was found. The reason for this situation may be that overexpression of *PTTG1* plays an important role in the occurrence of ESCC, but it may have little effect on the development and progression of ESCC. Of course, this hypothesis needs to be further verified by our future experiments. We did not establish a relationship between the high expression of *PTTG1* and other pathological parameters that represented the clinical process; however, the expression of *PTTG1* in patients less than or equal to 60 years old was higher than that of patients older than 60, and a higher expression of *PTTG1* was correlated to a lower age. The mechanism of the relationship between *PTTG1* and the patient’s age was unclear and requires further study.

We then investigated the reasons for the high expression of *PTTG1*. The mechanism of *PTTG1* in ESCC has been reported in previous studies. *PTTG1* induces EMT and metastasis in ESCC by activating GLI1 (Feng et al., 2017). *PTTG1* might also regulate the expression of multiple genes related to cell movement, including members of the Ras–Rho oncogene superfamily (Ito et al., 2008). As a transcription factor, *PTTG1* may achieve its biological function through targeted regulation of a certain set of genes. However, there was no report on the potential target genes of *PTTG1* in ESCC. By combining the target genes displayed by ChIP-seq of *PTTG1*, DEGs of ESCC, *PTTG1*-related genes, and the possible signaling pathways of *PTTG1*, it was observed that *PTTG1* may interact with these genes through chemokines and cytokine signaling pathways. The inflammatory response, integrin signaling pathway, ubiquitin proteasome pathway, and apoptosis signaling pathway played a role in ESCC. By constructing a PPI network and combining ChIP-seq data, we obtained four potential target genes, *SPTAN1*, *SLC25A17*, *IKBKB*, and *ERH*. These hub genes all had binding sites with *PTTG1*. Interestingly, these genes had been reported as tumor related, and *SPTAN1* was a family of filamentous cytoskeletal proteins that acted as an essential scaffold protein for stabilizing the plasma membrane and organizing intracellular organelles. The encoded protein had functions of DNA repair and cell cycle regulation. The expression of *SPTAN1* in metastatic colon cancer was lower than that in non-metastatic colon cancer. The low expression of *SPTAN1* reduced the ability of cell-to-cell contact, which facilitated the metastasis of colon cancer cells (Hinrichsen et al., 2014; Ackermann et al., 2019). It was found that *SPTAN1* expression increased in ovarian cancer after chemotherapy (L’Espérance et al., 2006). The *SLC25A17* gene was the only member of the mitochondrial carrier family that had previously been confirmed to be located in the peroxisomal membrane. It encoded a peroxisomal membrane protein of the mitochondrial solute carrier family and display varied expression levels in 21 human tissues (including brain, esophagus, lung, liver, and kidney tissues) (Agrimi et al., 2012). However, only one relevant study reported its role in tumors, showing that genetic changes in *SLC25A17* (CNV deletion) were closely associated with the overall survival rate and relapse-free survival rate of neuroblastoma patients (Khan et al., 2015). Because *SLC25A17* is positively correlated with TF *PTTG1* expression, it was worth further study. *IKBKB*, also known as IKKβ, is a nuclear factor κB kinase subunit β inhibitor, affecting cell physiology in many ways and phosphorylating proteins to regulate cellular processes from the cell cycle to metabolism and differentiation (Page et al., 2017). Excessive activity of *IKBKB* in oral and dental epithelial cells leads to changes in the composition of epithelial cells and the expression of several tumor suppressor proteins and miRNAs. This results in cells in a pre-tumor state, and the lack of p53 or p16 tumor suppressor proteins and the loss of p19 would cause odontogenic tumors and metastases to local lymph nodes and lungs. *IKBKB* was observed in the areas of tumor ameloblasts that express keratin K5 at high levels (Page et al., 2020). *IKBKB* could be activated by α-KG produced by *GDH1* and then upregulate *GLUT1* to promote glucose uptake and tumor cell survival, thus accelerating the occurrence of glioma (Wang et al., 2019). *ERH* was an mRNA splicing and mitotic factor, and its function was related to pyrimidine biosynthesis and cell cycles (Gelsthorpe et al., 1997). The *ERH* gene might affect the apoptosis of bladder cancer T24 cells through TLR, NF-κB, TNF, or TGF-β signaling pathways, activating the growth of malignant tumors (Pang et al., 2020). The research team also proved that *ERH* knockdown could inhibit the transfer of BUC T24 cells *in vitro* and *in vivo* through nude mice tail vein metastasis experiments. They found that *ERH* gene knockdown suppressed MYC-mediated migration and invasion in bladder cancer T24 cells (Pang et al., 2019). In primary human breast cancer and breast cancer cell models, quantitative RT-PCR found that *ERH* expression was significantly more upregulated in tumorigenic breast cancer cell lines than in non-tumorigenic cell lines (Zafrakas et al., 2008). This study also found a significant positive correlation between *ERH* and *PTTG1* expression; the molecular relationship between these is also worthy of further study. These reports, to some extent, supported our hypothesis that *PTTG1* may play an important role in the occurrence and development of ESCC by upregulating *SLC25A17* and *ERH*.

Future research could continue this work and improve on the current deficiencies. First, extra research should be conducted on the relationship between *PTTG1* and ESCC progress and prognosis to explore whether *PTTG1* could act as a factor to evaluate the prognosis of ESCC. Second, the research should investigate whether *PTTG1* can be detected in body fluids and whether it contributes to the early diagnosis of ESCC. Third, a ChIP-seq of *PTTG1* using the ESCC cell line needs to be performed. Although the target genes of *PTTG1* in different tissue cells might partially overlap, they also have tumor specificity. Fourth, *in vivo* and *in vitro* experiments need to be carried out to study the biological functions and related molecular mechanisms of *PTTG1*. Fifth, related experiments need to demonstrate the function of the highlighted potential markers in our study.

## Conclusion

This study combined multiple detection methods—IHC, RNA-seq, and gene chip data—to comprehensively demonstrate for the first time that *PTTG1* was highly expressed in ESCC tissues. This was true from the protein level to the mRNA level and from individual research to integrated analysis. The results were confirmed by a study based on 2,721 cases. It was found that the high expression of *PTTG1* may play an important role in the formation of ESCC. These roles may be completed by *PTTG1* regulating the downstream target genes *SLC25A17* and *ERH*; however, this hypothesis requires extra experiments to verify.

## Data Availability Statement

The data presented in the study are deposited in the Gene Expression Omnibus (GEO) (https://www.ncbi.nim.nih.gov/geo/) repository, accession number (GSE164158).

## Ethics Statement

The studies involving human participants were reviewed and approved by the First Affiliated Hospital of Guangxi Medical University. The patients/participants provided their written informed consent to participate in this study.

## Author Contributions

All authors contributed to the study conception and design. H-FZ performed the data extraction and statistical analysis and drafted the manuscript. S-WC and H-JZ conceived and designed the study and edited the manuscript. L-JY, R-QH, and GC contributed to the design of the study, supervised all the experiments, and corrected the manuscript. Z-GH, XY, Y-WD, JL, and Z-WF collected, extracted, and analyzed the data. J-XM, Z-QT, C-BL, RL, L-HY, and JM critically revised the manuscript. All authors read and approved the final manuscript.

## Conflict of Interest

The authors declare that the research was conducted in the absence of any commercial or financial relationships that could be construed as a potential conflict of interest.

## Footnotes

1 ftp://ftp.ensembl.org/pub/relase-87/fasta/homo_sapiens/dna/

2 http://cistrome.org/db/#/
