# Supplementary material for: The Clinical Significance and Potential Molecular Mechanism of PTTG1 in Esophageal Squamous Cell Carcinoma
Source: Front Genet. 2021 Jan 22;11:583085. doi: 10.3389/fgene.2020.583085 (PMC7863988; doi:10.3389/fgene.2020.583085)
Supplement: Supplementary file 1 [file Table_1.DOCX]

**Supplementary Table 1**. IHC scores of *PTTG1* in each ESCC and normal cases based on two different pathologists, separately.

| Tumor case |  | IHC score | |  | Normal case |  | IHC score | |
| --- | --- | --- | --- | --- | --- | --- | --- | --- |
|  |  | Pathologist 1 | Pathologist 2 |  |  |  | Pathologist 1 | Pathologist 2 |
| T1 |  | 12 | 12 |  | N1 |  | 8 | 8 |
| T2 |  | 12 | 12 |  | N2 |  | 8 | 8 |
| T3 |  | 12 | 12 |  | N3 |  | 8 | 8 |
| T4 |  | 9 | 9 |  | N4 |  | 6 | 6 |
| T5 |  | 9 | 9 |  | N5 |  | 9 | 9 |
| T6 |  | 8 | 8 |  | N6 |  | 6 | 6 |
| T7 |  | 12 | 12 |  | N7 |  | 6 | 6 |
| T8 |  | 9 | 9 |  | N8 |  | 6 | 6 |
| T9 |  | 9 | 9 |  | N9 |  | 6 | 6 |
| T10 |  | 12 | 12 |  | N10 |  | 12 | 12 |
| T11 |  | 12 | 12 |  | N11 |  | 8 | 8 |
| T12 |  | 6 | 6 |  | N12 |  | 8 | 8 |
| T13 |  | 8 | 8 |  | N13 |  | 6 | 6 |
| T14 |  | 12 | 12 |  | N14 |  | 6 | 6 |
| T15 |  | 8 | 8 |  | N15 |  | 8 | 8 |
| T16 |  | 8 | 8 |  | N16 |  | 9 | 9 |
| T17 |  | 12 | 12 |  | N17 |  | 12 | 8 |
| T18 |  | 12 | 8 |  | N18 |  | 8 | 8 |
| T19 |  | 12 | 8 |  | N19 |  | 8 | 8 |
| T20 |  | 8 | 8 |  | N20 |  | 9 | 9 |
| T21 |  | 12 | 12 |  | N21 |  | 12 | 12 |
| T22 |  | 8 | 8 |  | N22 |  | 8 | 8 |
| T23 |  | 12 | 12 |  | N23 |  | 8 | 8 |
| T24 |  | 12 | 12 |  | N24 |  | 6 | 6 |
| T25 |  | 9 | 9 |  | N25 |  | 6 | 6 |
| T26 |  | 12 | 12 |  | N26 |  | 8 | 8 |
| T27 |  | 12 | 12 |  | N27 |  | 8 | 8 |
| T28 |  | 12 | 12 |  | N28 |  | 8 | 8 |
| T29 |  | 12 | 12 |  | N29 |  | 8 | 8 |
| T30 |  | 12 | 12 |  | N30 |  | 6 | 6 |
| T31 |  | 12 | 12 |  | N31 |  | 6 | 6 |
| T32 |  | 9 | 9 |  | N32 |  | 6 | 6 |
| T33 |  | 9 | 9 |  | N33 |  | 6 | 6 |
| T34 |  | 12 | 12 |  | N34 |  | 6 | 6 |
| T35 |  | 12 | 12 |  | N35 |  | 6 | 6 |
| T36 |  | 8 | 8 |  | N36 |  | 6 | 6 |
| T37 |  | 9 | 9 |  | N37 |  | 9 | 9 |
| T38 |  | 12 | 12 |  | N38 |  | 8 | 8 |
| T39 |  | 9 | 9 |  | N39 |  | 6 | 6 |
| T40 |  | 12 | 12 |  | N40 |  | 8 | 8 |
| T41 |  | 12 | 12 |  | N41 |  | 8 | 8 |
| T42 |  | 8 | 8 |  | N42 |  | 4 | 4 |
| T43 |  | 8 | 8 |  | N43 |  | 8 | 8 |
| T44 |  | 8 | 8 |  | N44 |  | 8 | 8 |
| T45 |  | 12 | 12 |  | N45 |  | 8 | 8 |
| T46 |  | 12 | 12 |  | N46 |  | 8 | 8 |
| T47 |  | 12 | 12 |  | N47 |  | 4 | 4 |
| T48 |  | 12 | 12 |  | N48 |  | 8 | 8 |
| T49 |  | 12 | 12 |  | N49 |  | 4 | 4 |
| T50 |  | 12 | 12 |  | N50 |  | 8 | 8 |
| T51 |  | 12 | 12 |  | N51 |  | 12 | 12 |
| T52 |  | 8 | 8 |  | N52 |  | 9 | 9 |
| T53 |  | 12 | 8 |  | N53 |  | 4 | 4 |
| T54 |  | 12 | 12 |  | N54 |  | 8 | 4 |
| T55 |  | 12 | 12 |  | N55 |  | 4 | 4 |
| T56 |  | 12 | 12 |  | N56 |  | 8 | 8 |
| T57 |  | 12 | 12 |  | N57 |  | 8 | 8 |
| T58 |  | 12 | 12 |  | N58 |  | 8 | 8 |
| T59 |  | 12 | 12 |  | N59 |  | 8 | 8 |
| T60 |  | 12 | 12 |  | N60 |  | 8 | 8 |
| T61 |  | 9 | 9 |  | N61 |  | 8 | 8 |
| T62 |  | 12 | 12 |  | N62 |  | 8 | 8 |
| T63 |  | 8 | 8 |  | N63 |  | 8 | 4 |
| T64 |  | 9 | 9 |  | N64 |  | 6 | 6 |
| T65 |  | 8 | 8 |  | N65 |  | 6 | 6 |
| T66 |  | 8 | 8 |  | N66 |  | 6 | 6 |
| T67 |  | 12 | 12 |  | N67 |  | 8 | 8 |
| T68 |  | 12 | 12 |  | N68 |  | 8 | 8 |
| T69 |  | 8 | 8 |  | N69 |  | 6 | 6 |
| T70 |  | 9 | 9 |  | N70 |  | 4 | 4 |
| T71 |  | 8 | 8 |  | N71 |  | 8 | 8 |
| T72 |  | 12 | 12 |  | N72 |  | 8 | 8 |
| T73 |  | 12 | 12 |  | N73 |  | 12 | 12 |
| T74 |  | 12 | 12 |  | N74 |  | 12 | 12 |
| T75 |  | 12 | 12 |  | N75 |  | 8 | 8 |
| T76 |  | 12 | 12 |  | N76 |  | 12 | 12 |
| T77 |  | 12 | 12 |  | N77 |  | 8 | 8 |
| T78 |  | 12 | 12 |  | N78 |  | 8 | 8 |
| T79 |  | 12 | 12 |  | N79 |  | 4 | 4 |
| T80 |  | 12 | 12 |  | N80 |  | 8 | 8 |
| T81 |  | 8 | 8 |  | N81 |  | 8 | 8 |
| T82 |  | 12 | 12 |  | N82 |  | 8 | 8 |
| T83 |  | 12 | 12 |  | N83 |  | 6 | 6 |
| T84 |  | 12 | 12 |  | N84 |  | 8 | 8 |
| T85 |  | 8 | 8 |  | N85 |  | 6 | 6 |
| T86 |  | 12 | 12 |  | N86 |  | 4 | 4 |
| T87 |  | 12 | 12 |  | N87 |  | 8 | 8 |
| T88 |  | 6 | 6 |  | N88 |  | 8 | 8 |
| T89 |  | 9 | 9 |  | N89 |  | 8 | 8 |
| T90 |  | 12 | 12 |  | N90 |  | 8 | 8 |
| T91 |  | 8 | 8 |  | N91 |  | 6 | 6 |
| T92 |  | 8 | 8 |  | N92 |  | 2 | 2 |
| T93 |  | 8 | 8 |  | N93 |  | 9 | 9 |
| T94 |  | 8 | 8 |  | N94 |  | 12 | 12 |
| T95 |  | 12 | 12 |  | N95 |  | 9 | 9 |
| T96 |  | 12 | 12 |  | N96 |  | 9 | 9 |
| T97 |  | 12 | 12 |  | N97 |  | 8 | 8 |
| T98 |  | 12 | 12 |  | N98 |  | 8 | 8 |
| T99 |  | 9 | 9 |  | N99 |  | 6 | 6 |
| T100 |  | 8 | 8 |  | N100 |  | 8 | 8 |
| T101 |  | 12 | 12 |  | N101 |  | 8 | 8 |
| T102 |  | 8 | 8 |  | N102 |  | 2 | 2 |
| T103 |  | 9 | 9 |  | N103 |  | 4 | 4 |
| T104 |  | 9 | 9 |  | N104 |  | 8 | 8 |
| T105 |  | 9 | 9 |  | N105 |  | 8 | 8 |
| T106 |  | 9 | 9 |  | N106 |  | 9 | 9 |
| T107 |  | 9 | 9 |  | N107 |  | 6 | 6 |
| T108 |  | 9 | 9 |  | N108 |  | 6 | 6 |
| T109 |  | 9 | 9 |  | N109 |  | 8 | 8 |
| T110 |  | 9 | 9 |  | N110 |  | 9 | 9 |
| T111 |  | 12 | 12 |  | N111 |  | 9 | 9 |
| T112 |  | 12 | 12 |  | N112 |  | 8 | 8 |
| T113 |  | 9 | 9 |  | N113 |  | 8 | 8 |
| T114 |  | 8 | 8 |  | N114 |  | 6 | 6 |
| T115 |  | 8 | 8 |  | N115 |  | 9 | 9 |
| T116 |  | 9 | 9 |  | N116 |  | 4 | 4 |
| T117 |  | 12 | 12 |  | N117 |  | 6 | 6 |
| T118 |  | 6 | 6 |  | N118 |  | 9 | 9 |
| T119 |  | 8 | 8 |  | N119 |  | 8 | 8 |
| T120 |  | 12 | 12 |  | N120 |  | 8 | 8 |
| T121 |  | 12 | 12 |  | N121 |  | 9 | 9 |
| T122 |  | 8 | 8 |  | N122 |  | 4 | 4 |
| T123 |  | 12 | 12 |  | N123 |  | 8 | 8 |
| T124 |  | 8 | 8 |  | N124 |  | 4 | 4 |
| T125 |  | 8 | 8 |  | N125 |  | 4 | 4 |
| T126 |  | 8 | 8 |  | N126 |  | 9 | 9 |
| T127 |  | 12 | 12 |  | N127 |  | 9 | 9 |
| T128 |  | 12 | 12 |  | N128 |  | 6 | 6 |
| T129 |  | 12 | 12 |  | N129 |  | 12 | 12 |
| T130 |  | 8 | 8 |  | N130 |  | 9 | 9 |
| T131 |  | 12 | 8 |  | N131 |  | 6 | 6 |
| T132 |  | 8 | 8 |  | N132 |  | 8 | 8 |
| T133 |  | 8 | 8 |  | N133 |  | 8 | 8 |
| T134 |  | 12 | 12 |  | N134 |  | 12 | 12 |
| T135 |  | 12 | 12 |  | N135 |  | 8 | 8 |
| T136 |  | 8 | 8 |  | N136 |  | 8 | 8 |
| T137 |  | 8 | 8 |  | N137 |  | 8 | 8 |
| T138 |  | 12 | 12 |  | N138 |  | 8 | 8 |
| T139 |  | 12 | 12 |  | N139 |  | 12 | 12 |
| T140 |  | 12 | 12 |  | N140 |  | 2 | 2 |
| T141 |  | 12 | 12 |  | N141 |  | 2 | 2 |
| T142 |  | 12 | 12 |  | N142 |  | 2 | 2 |
| T143 |  | 12 | 12 |  | N143 |  | 6 | 6 |
| T144 |  | 12 | 12 |  |  |  |  |  |
| T145 |  | 12 | 12 |  |  |  |  |  |
| T146 |  | 9 | 9 |  |  |  |  |  |
| T147 |  | 12 | 12 |  |  |  |  |  |
| T148 |  | 12 | 12 |  |  |  |  |  |
| T149 |  | 12 | 12 |  |  |  |  |  |
| T150 |  | 12 | 12 |  |  |  |  |  |
| T151 |  | 12 | 12 |  |  |  |  |  |
| T152 |  | 12 | 12 |  |  |  |  |  |
| T153 |  | 9 | 9 |  |  |  |  |  |
| T154 |  | 9 | 9 |  |  |  |  |  |
| T155 |  | 9 | 9 |  |  |  |  |  |
| T156 |  | 12 | 12 |  |  |  |  |  |
| T157 |  | 9 | 9 |  |  |  |  |  |
| T158 |  | 12 | 12 |  |  |  |  |  |
| T159 |  | 8 | 8 |  |  |  |  |  |

ESCC, esophageal squamous cell carcinoma; IHC, immunohistochemistry; T, tumor; N, Normal.
