# Supplementary material for: The Clinical Significance and Potential Molecular Mechanism of PTTG1 in Esophageal Squamous Cell Carcinoma
Source: Front Genet. 2021 Jan 22;11:583085. doi: 10.3389/fgene.2020.583085 (PMC7863988; doi:10.3389/fgene.2020.583085)
Supplement: Supplementary file 2 [file Table_2.DOCX]

**Supplementary table 2**. The relationship between *PTTTG1* protein expression levels and clinical pathological features based on in-house ESCC tissue microarrays.

| Parameters | N | Mean | SD | P |
| --- | --- | --- | --- | --- |
| Tissue |  |  |  | <0.001 |
| Tumor | 159 | 10.410 | 1.880 |  |
| Control | 143 | 7.410 | 2.153 |  |
| Gender |  |  |  | 0.549 |
| Male | 144 | 10.440 | 1.835 |  |
| Female | 15 | 10.070 | 2.314 |  |
| Age |  |  |  | 0.908 |
| ≤60 | 97 | 10.420 | 1.875 |  |
| >60 | 62 | 10.390 | 1.902 |  |
| T |  |  |  | 0.104 |
| T1-2 | 52 | 10.750 | 1.792 |  |
| T3-4 | 107 | 10.240 | 1.907 |  |
| N |  |  |  | 0.406 |
| N0 | 73 | 10.270 | 1.865 |  |
| N1-N3 | 86 | 10.520 | 1.896 |  |
| TNM |  |  |  | 0.798 |
| I-II | 88 | 10.440 | 1.844 |  |
| III-IV | 71 | 10.370 | 1.936 |  |

Note: T: tumor pathological stage; N: node pathological stage; SD: standard deviation; M: metastasis pathological stage; TNM: pathological TNM stage.
