# Supplementary material for: The Clinical Significance and Potential Molecular Mechanism of PTTG1 in Esophageal Squamous Cell Carcinoma
Source: Front Genet. 2021 Jan 22;11:583085. doi: 10.3389/fgene.2020.583085 (PMC7863988; doi:10.3389/fgene.2020.583085)
Supplement: Supplementary file 3 [file Table_3.DOCX]

**Supplementary Table 3**. The relationship between *PTTTG1* mRNA expression levels and clinical pathological features based on TCGA database.

| Parameters | N | Mean | SD | P |
| --- | --- | --- | --- | --- |
| Tissue |  |  |  | <0.001 |
| Tumor | 82 | 11.579 | 0.727 |  |
| Control | 1456 | 7.558 | 2.513 |  |
| Gender |  |  |  | 0.110 |
| Male | 69 | 11.637 | 0.702 |  |
| Female | 12 | 11.272 | 0.843 |  |
| Age |  |  |  | 0.004 |
| ≤60 | 52 | 11.755 | 0.726 |  |
| >60 | 29 | 11.274 | 0.642 |  |
| Alcohol |  |  |  | 0.632 |
| NO | 19 | 11.656 | 0.884 |  |
| YES | 60 | 11.562 | 0.690 |  |
| T |  |  |  | 0.369 |
| T1-2 | 36 | 11.501 | 0.812 |  |
| T3-4 | 45 | 11.649 | 0.660 |  |
| N |  |  |  | 0.169 |
| N0 | 43 | 11.466 | 0.692 |  |
| N1-N3 | 30 | 11.697 | 0.708 |  |
| M |  |  |  | 0.258 |
| M0 | 70 | 11.575 | 0.768 |  |
| M1 | 5 | 11.970 | 0.230 |  |
| TNM |  |  |  | 0.275 |
| I-II | 54 | 11.526 | 0.711 |  |
| III-IV | 26 | 11.718 | 0.776 |  |
| Grade |  |  |  | 0.590 |
| I-II | 53 | 11.655 | 0.730 |  |
| III-IV | 19 | 11.545 | 0.824 |  |

Note: T: tumor pathological stage; N: node pathological stage; SD: standard deviation; M: metastasis pathological stage; TNM: pathological TNM stage.
