# Supplementary figures and images for: The Clinical Significance and Potential Molecular Mechanism of PTTG1 in Esophageal Squamous Cell Carcinoma
Source: Front Genet. 2021 Jan 22;11:583085. doi: 10.3389/fgene.2020.583085 (PMC7863988; doi:10.3389/fgene.2020.583085)

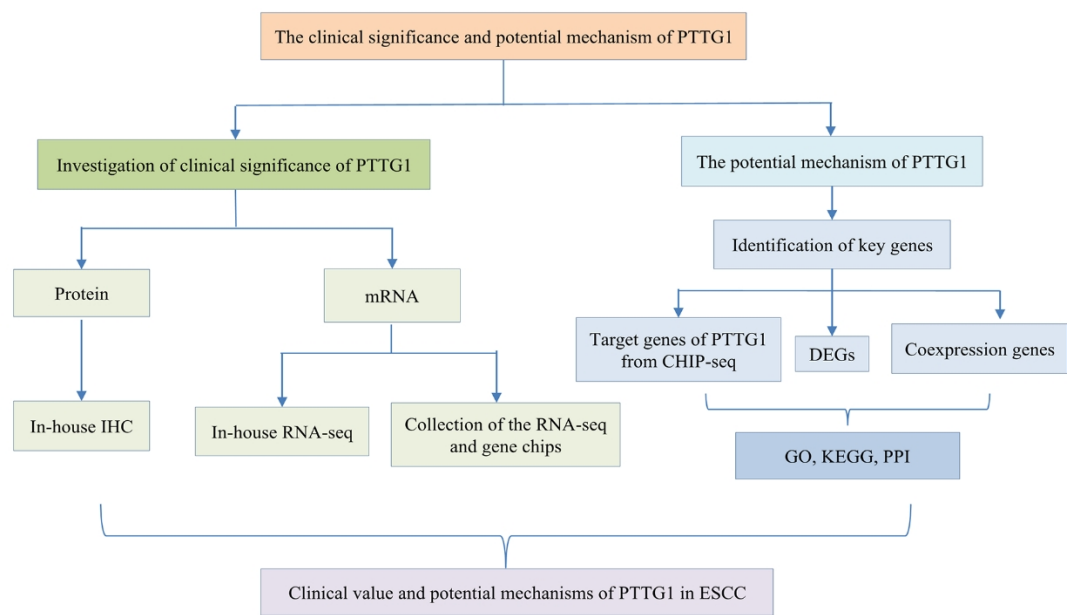

Supplementary Figure 1. The overall flow chart of this study.

Supplement: Supplementary file 4 [file Data_Sheet_1.PDF]
