# Supplementary material for: The Clinical Significance and Potential Molecular Mechanism of PTTG1 in Esophageal Squamous Cell Carcinoma
Source: Front Genet. 2021 Jan 22;11:583085. doi: 10.3389/fgene.2020.583085 (PMC7863988; doi:10.3389/fgene.2020.583085)
Supplement: Supplementary file 5 [file Data_Sheet_2.PDF]

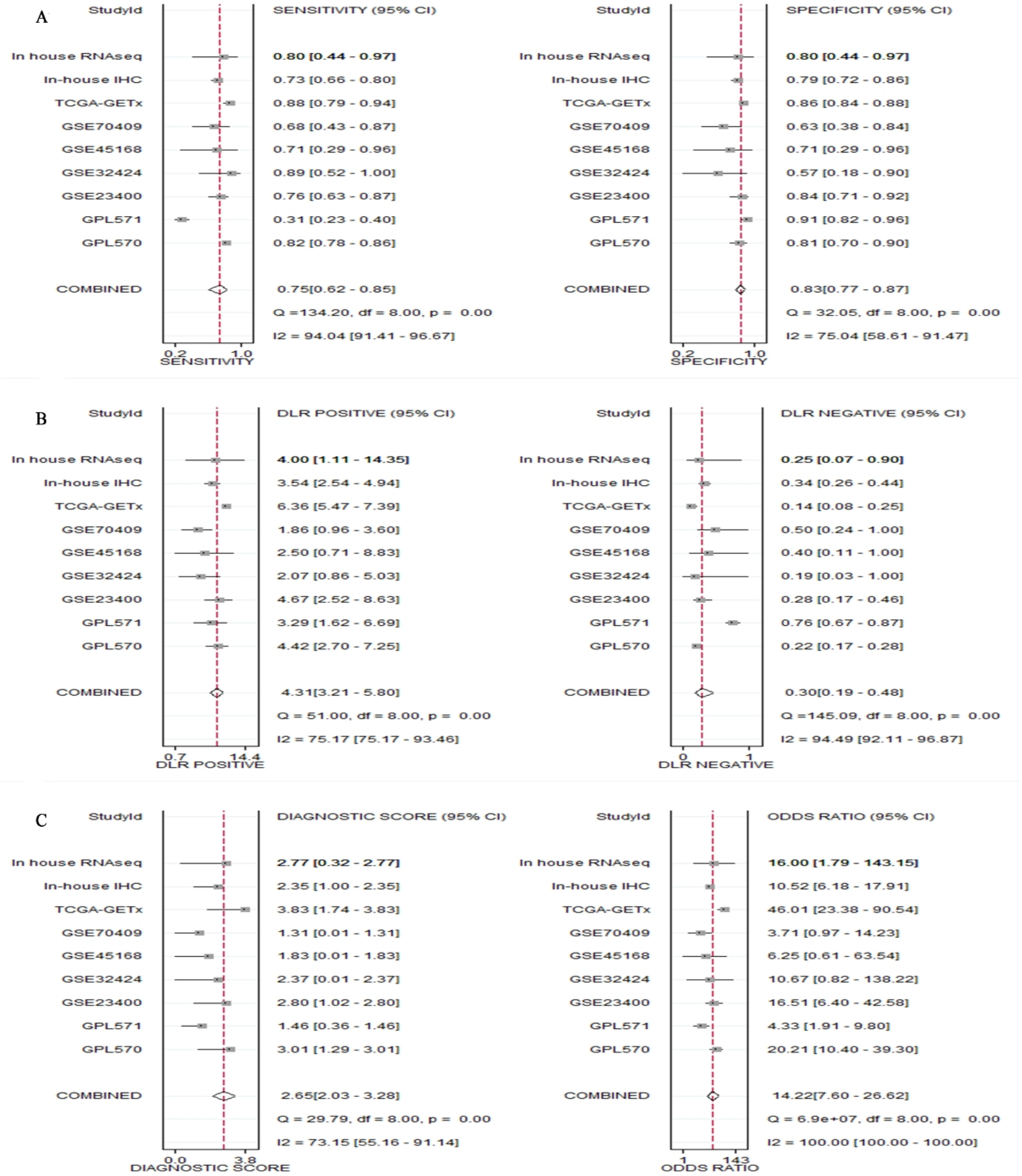

Supplementary Figure 2. Integrated analysis of all data sets for diagnostic ability of *PTTG1* expression. (A) Sensitivity and specificity forest map; (B) Positive likelihood ratio and negative likelihood ratio forest diagram; (C) Diagnostic advantage ratio forest diagram.
